# Circulating levels of DLK1 and glucose homeostasis in girls with obesity: A pilot study

**DOI:** 10.3389/fendo.2022.1033179

**Published:** 2022-12-09

**Authors:** Stefania Palumbo, Giuseppina Rosaria Umano, Francesca Aiello, Grazia Cirillo, Emanuele Miraglia del Giudice, Anna Grandone

**Affiliations:** Department of the Woman, of the Child, of General and Specialized Surgery, University of Campania “Luigi Vanvitelli”, Naples, Italy

**Keywords:** Dlk1, insulin-resistance, children and adolescents, obesity, adipose tissue

## Abstract

**Introduction:**

DLK1 gene is considered a molecular gatekeeper of adipogenesis. DLK1 mutations have been reported as a cause of central precocious puberty associated with obesity and metabolic syndrome with undetectable DLK1 serum levels. We investigated the association between DLK1 circulating levels with clinical and biochemical parameters in obese adolescents and healthy controls.

**Methods:**

Sixty-five obese adolescents and 40 controls were enrolled and underwent a complete clinical examination and biochemical assessment for glucose homeostasis and DLK1 plasma levels.

**Results:**

We observed lower DLK1 levels in cases compared to controls. Moreover, we found a negative correlation between DLK1 and HOMA-IR and a direct correlation with insulin-sensitivity index.

**Discussion:**

Our findings suggest that DLK1 might be involved in metabolic derangement in obese children.

## Introduction

DLK1 (Delta-like 1 homolog) is a membrane-bound protein that plays an important role in inhibiting adipocyte differentiation (1, 2). It is part of the Notch signalling pathway controlling many developmental processes also having neuroendocrine function, suggested by its postnatal expression in hypothalamic nuclei (3, 4). It is located on chromosome 14 in the imprinted region 14q32, whose maternal uniparental disomy causes Temple’s syndrome, a condition characterized by hypotonia, prenatal growth failure, short postnatal stature, early puberty, and truncal obesity (5). Moreover, mutations in *DLK1* gene have been reported as a cause of central precocious puberty associated with obesity and metabolic syndrome with undetectable DLK1 serum levels (6). Animal model studies have shown that *DLK1* knockout mice exhibit growth retardation and obesity (7), while *DLK1* overexpression leads to decreased fat mass, diet-induced obesity resistance and reduced insulin signalling (8–10). In line with these data, deficiency of DLK1 in humans, both for imprinting defects such as in Temple syndrome and in cases of mutations in the gene, is associated with undetectable DLK1 levels and childhood and adolescent obesity.

The aim of the present preliminary study is to investigate circulating levels of DLK1 within the context of human paediatric obesity and its relationship with clinical and biochemical parameters.

## Materials and methods

The study was conducted at paediatric endocrinology clinic of University of Campania Luigi Vanvitelli of Naples, Italy. Considering the relationship between DLK1 and puberty and the possible sex dependent nature of this relationship, we focused only on girls in this pilot study, also as the completion of pubertal development through registering age at menarche can be assessed with greater certainty in girls compared to boys. We enrolled 65 girls with obesity (mean age:12.3 ± 5.5; BMI z-score:2.9 ± 0.8) with normal puberty onset time (thelarche >8 years or age at menarche >10 years) and a body mass index (BMI) above the 95th percentile and 40 pubertal stage-matched control female patients (mean age:12.1 ± 1.2 BMI z-score: -0.6 ± 1.2). Clinical examination was performed in all girls, including weight and height measurement, and BMI z-score calculation according to the LMS (least mean squares) method. All blood samples were drawn at 8:00 a.m. from an antecubital vein, clotted, centrifuged, and serum was stored at −20°C until analyses were performed.

Fasting samples for glucose, insulin, triglycerides, total cholesterol, high-density lipoprotein cholesterol (HDLC), serum aspartate transaminase (AST) and alanine transaminase (ALT) were obtained. All subjects with obesity underwent a standard two‐hour oral glucose tolerance test administrating 1,75g/kg glucose up to 75gr orally. Blood samples for plasma glucose and insulin were obtained every 30 minutes. Indexes of insulin-resistance (homeostasis model assessment of insulin resistance, HOMA-IR, and whole-body insulin sensitivity index, WBISI) were calculated as previously described (11).

Serum Dlk1 concentrations were determined using the commercially available Human DLK1 ELISA

(MyBioSource, San Diego, CA, USA) with a detection limit of 0.216 ng/mL. Intra-assay and inter-assay coefficients of variation (CVs) listed by the manufacturer were of <10 and <12%, respectively.

Continuous variables were checked for normality according to the Kolmogorov-Smirnov test. Differences for continuous variables were investigated with Student t-test for independent samples and Mann-Whitney U test as appropriate. Chi Square test was performed to test differences in categorical variables. Spearman correlation analyses were performed to test the correlation between DLK1 levels and insulin-resistance measures. Data are expressed as mean and standard deviation or median and interquartile range according to normal or not normal distribution.

Hepatic steatosis was defined as present or absent according to abdominal ultrasound. It was assessed according to abnormally intense echoes arising from the hepatic parenchyma, and liver-kidney differences in echo amplitude. Two experienced radiologists performed the ultrasound for hepatic steatosis detection.

## Results

**Figure 1:**
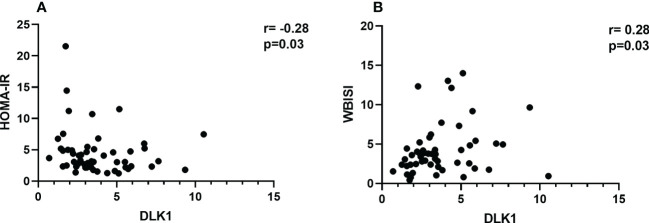
Spearman correlation analyses between DLK1 levels and insulin resistance measures in girls with obesity. **(A)** displays the correlation between DLK1 and HOMA-IR. **(B)** refers to the correlation between DLK1 and WBISI. Spearman correlation coefficient (rho, r) and significance level of the test statistic (p-value, p) are reported. Legend: HOMA-IR, homeostasis model assessment of insulin resistance; WBISI, whole-body insulin sensitivity index.

The anthropometric and biochemical characteristics of the cohort are reported in (**Table 1**). The two groups did not differ in age distribution (12.1 ± 1.2 in girls with obesity and 12.3 ± 2.5 in control group, p=0.76) and pubertal stage (21% prepubertal children in control group and 16% in girls with obesity, p=0.57). As expected, the group with obesity showed significantly higher z-score BMI compared to controls (p<0.0001). The results obtained from the serum assay of circulating protein revealed lower levels in the group of subjects with obesity with a median of 3.36 ng/ml (IQR 3.10) compared with the group of healthy patients with a median of 4.58 ng/ml (IQR 1.88, p=0.01). A significant negative correlation between DLK1 levels and HOMA-IR was also observed (r=-0.28; p= 0.03) (**Figure 1A**) while a direct correlation was found with insulin sensitivity index (WBISI) (r=0.28; p=0.03) (**Figure 1B**). Four out 65 (6%) obese girls showed prediabetes (1 IFG and 3 IGT). DLK1 levels were significantly lower in girls with prediabetes compared to normoglycemic girls (p=0.02). Abdominal ultrasound was available for 53 out 65 obese girls. Among them, NAFLD was present in 39.6% of cases. No difference for DLK1 levels were found between girls with NAFLD and girls without NAFLD.

**Table 1:** Clinical and laboratory features of the study cohort.

Parameters	Ctrl	OB	p
**N**	40	65	
**Age (y)**	12.1 ± 1.2	12.3 ± 2.5	0.76
**z-score BMI**	-0.6 ± 1.2	2.9 ± 0.8	**<0.0001**
**Age of Menarche (y)**	11.5 ± 0.6	11.2 ± 1	0.50
**LDL-cholesterol (mg/dl)**	-	93.1 ± 29	
**HDL-cholesterol (mg/dl)**	**-**	44.4 ± 9.9	
**Fasting glucose (mg/dl)**	77.0 ± 6.1	77.7 ± 10.8	0.82
**Fasting Insulin (mcU/ml)**	–	23.7 ± 20.4	
**HOMA-IR**	–	4.4 ± 3.6	
**WBISI**	–	4.8 ± 4.6	
**DLK1 (ng/ml)**	4.58 (IQR 1.88)	3.36 (IQR 3.10)	**0.01**

All the values are expressed as mean ± SD. Significant differences are in bold. Ctrl, controls; HOMA-IR, homeostasis model assessment of insulin resistance; IQR, interquartile range; N, number; OB, patients with obesity; WBISI, whole-body insulin sensitivity index.

## Discussion

Our study reported lower serum levels of DLK1 in subjects with obesity suggesting its role in the regulation of adiposity even in the absence of syndromic condition or precocious puberty.

The role of DLK1 in obesity is not completely clear but studies in animal models have shown that DLK1 may play a role not only in adipogenesis but also in adaptive thermogenesis in adipose tissue, the phenomenon also called browning (12). Future studies focusing on the role of DLK1 in this process in humans would be warranted, as it could represent an interesting target for the treatment of obesity and its complications. Moreover, data about correlation between Dlk1 levels and both body fat percentage and insulin resistance are conflicting. In the present study has been observed a weak, albeit statistically significant, negative correlation between DLK1 serum levels and insulin-resistance degree in girls with obesity. This data is in line with those observed by Demir Çalteki et al. in a cohort of women with polycystic ovary syndrome (PCOS) (13) with low DLK1 levels and an inverse correlation between HOMA-IR and DLK1. In addition, a previous study revealed lower DLK1 serum concentrations in patients with obesity and Type 2 Diabetes (T2D) compared to non-T2D subjects (14). A longitudinal study on a large cohort of adult diabetic patients showed significantly lower levels of DLK1 in 4 individuals with increased fasting plasma glucose or whose homeostasis model assessment of β-cell function (HOMA-β) was decreased at the follow-up compared to the control group. Notably, these results were significant in women but not in men (15). Also, studies on animal models reported how DLK1 can influence fat and glucose metabolism in the liver. In particular, DLK1 administration in mice promotes hepatic fatty acid oxidation and inhibits gluconeogenesis (16). These findings supported the hypothesis that DLK1 plays a role in glucose/insulin homeostasis. In particular, scientific evidence indicates that DLK1 inhibits Notch1 function. Notch activation pathologically affects lipogenesis and gluconeogenesis, finally increasing insulin-resistance (16). Therefore, the DLK1 inhibitory activity on Notch1 signaling might mediate the modulatory effect of DLK1 on glucose metabolism.

Paternally transmitted foetal DLK1 genotype affected maternal DLK1 levels that were positively associated with insulin-resistance and inversely correlated with insulin secretion during the third trimester of pregnancy (17). In contrast, other studies have reported that DLK1 is negatively associated with insulin sensitivity during pregnancy and in non-diabetic men (18). In diabetic men, DLK1 was shown to reduce skeletal muscle glucose uptake without affecting hepatic gluconeogenesis (18). Similarly, in a cohort of pre-pubertal Spanish children DLK1 serum levels were positively correlated with plasma insulin, HOMA-IR, and free fatty acids (19). This effect was dependent on changes in dehydroepiandrosterone-sulphate (DHEA-S) levels suggesting a reciprocal influence of adrenal hormones and DLK1 on adrenal gland function and metabolic control (19). These contradictory findings highlight the need to further investigate the role of DLK1 in glucose homeostasis. The dynamic expression of DLK1 and the controversies in literature findings can be explained by the heterogeneity of the individuals included in the several studies in terms of age, pubertal stage, gender, comorbidities (diabetes, obesity, cardiovascular diseases), or other variables. Some reports have reported that sexual hormones influence DLK1 levels. The hypothesis of a hormonal influence might partly explain the different results reported in literature (20, 21). Therefore, considering the imprinted nature of *DLK1* gene, a sexual dimorphism of DLK1 effects might be speculated.

This study presents several limitations that should be acknowledged, such as the small sample size and the lack of data on HOMA-IR and WBISI in the non-obese controls.

In conclusion, preliminary data obtained in the present study show lower DLK1 serum levels in subjects with obesity compared to lean controls. Although we report a weak correlation with insulin resistance, this finding would suggest a DLK1-mediated metabolic effect. Confirmation of this result on a larger population would allow to add circulating DLK1 level assessment as a new marker of metabolic alterations in adolescents with obesity. New studies will be needed to further investigate and confirm these data and to clarify in humans the role of DLK1 in insulin secretion, adipogenesis and in the browning process.

## Data availability statement

The raw data supporting the conclusions of this article will be made available by the authors, without undue reservation.

## Ethics statement

The studies involving human participants were reviewed and approved by Ethic Commitee of the University of Campania Luigi Vanvitelli. Written informed consent to participate in this study was provided by the participants’ legal guardian/next of kin.

## Author contributions

SP: Writing - original draft, investigation; GU: Formal analysis, FA: Data curation; GC: Project administration, methodology; EG: Writing - review & editing; AG: Conceptualization, Resources, Supervision. All authors contributed to the article and approved the submitted version.
